# Strain-affected ferroelastic domain walls in RbMnFe charge-transfer materials undergoing collective Jahn–Teller distortion

**DOI:** 10.1039/d4ra06397j

**Published:** 2024-11-04

**Authors:** Marius Hervé, Shintaro Akagi, Laurent Guérin, Leland B. Gee, Ryan D. Ribson, Matthieu Chollet, Marco Cammarata, Shuntaro Nagashima, Shin-ichi Ohkoshi, Hiroko Tokoro, Eric Collet

**Affiliations:** a Univ Rennes, CNRS, IPR (Institut de Physique de Rennes) – UMR 6251 35000 Rennes France marius.herve@univ-rennes.fr eric.collet@univ-rennes.fr; b CNRS, Univ Rennes, DYNACOM (Dynamical Control of Materials Laboratory) – IRL 2015, The University of Tokyo 7-3-1 Hongo Tokyo 113-0033 Japan; c Department of Materials Science, Faculty of Pure and Applied Sciences, University of Tsukuba 1-1-1 Tennodai Tsukuba Ibaraki 305-8577 Japan; d Linac Coherent Light Source, SLAC National Accelerator Laboratory Menlo Park CA USA; e ESRF – The European Synchrotron 71 Avenue des Martyrs, CS40220 38043 Grenoble Cedex 9 France; f Department of Chemistry, School of Science, The University of Tokyo 7-3-1 Hongo, Bunkyo-ku Tokyo 113-0033 Japan; g Institut Universitaire de France (IUF) 75231 Paris France

## Abstract

Many rubidium manganese hexacyanoferrate materials, with the general formula Rb_*x*_Mn[Fe(CN)_6_]_(*x*+2)/3_·*z*H_2_O, exhibit diverse charge-transfer-based functionalities due to the bistability between a high temperature Mn^II^(*S* = 5/2)Fe^III^(*S* = 1/2) cubic phase and a low-temperature Mn^III^(*S* = 2)Fe^II^(*S* = 0) tetragonal phase. The collective Jahn–Teller distortion on the Mn sites is responsible for the cubic-to-tetragonal ferroelastic phase transition, which is associated with the appearance of ferroelastic domains. In this study, we use X-ray diffraction to reveal the coexistence of 3 types of ferroelastic tetragonal domains and estimate the spatial extension of the strain around the domain walls, which represents about 30% of the volume of the crystal.

## Abstract 2

This X-ray diffraction study of RbMnFe materials reveals the ferroelastic domains walls resulting from collective Jahn–Teller distortion coupled to intermetallic charge-transfer and the spatial extension of the strain around the walls.

## Graphical abstract

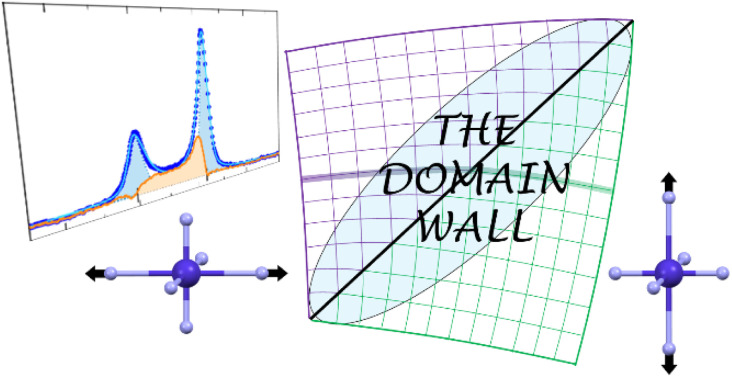

## Introduction

Many materials exhibit a ferroelastic phase transition,^1–3^ where the symmetry-breaking between different crystalline systems induces a spontaneous strain in the low symmetry phase. Ferroelastic materials show rich properties, with important applications in memory, multifunctional and novel controllable devices. In this respect, the cubic–tetragonal ferroelastic distortion was deeply investigated in many systems,^4–9^ with detailed analysis of the associated volume and tetragonal distortion strains. When such a symmetry breaking occurs, ferroelastic domains usually form,^10^ separated by a domain wall. The domain wall represents a twin interface where a multitude of properties can emerge.^11^

There are many switchable magnetic and molecular-based materials, which exhibit ferroelastic phase transitions coupled to an electronic bistability, such as spin-crossover (SCO) or charge-transfer (CT) in cyanide-bridge heterobimetallic materials.^12–20^ The effect of this coupling between both phenomena was rationalized within the frame of Collet's approach of the Landau theory of phase transition,^21^ which successfully explained the simultaneous or the sequential occurrence of electronic state switching and symmetry-breaking phenomena, the occurrence of hysteretic behaviour, and the emergence of various types of functions,^22–33^ including magnetoelectric (ME) effects and magnetic-field-induced spin state trapping (MIESST).^34–40^ The role of the ferroelastic domain walls and their dynamics was also scrutinized in Mn-based spin-crossover materials.^12–15^ It is now well understood that local properties can change around the deformed domain walls, giving rise for example to an electric polarization in an otherwise centrosymmetric system. The concept of functional domain walls opens new potential avenues for device designs.^11^

**Fig. 1:**
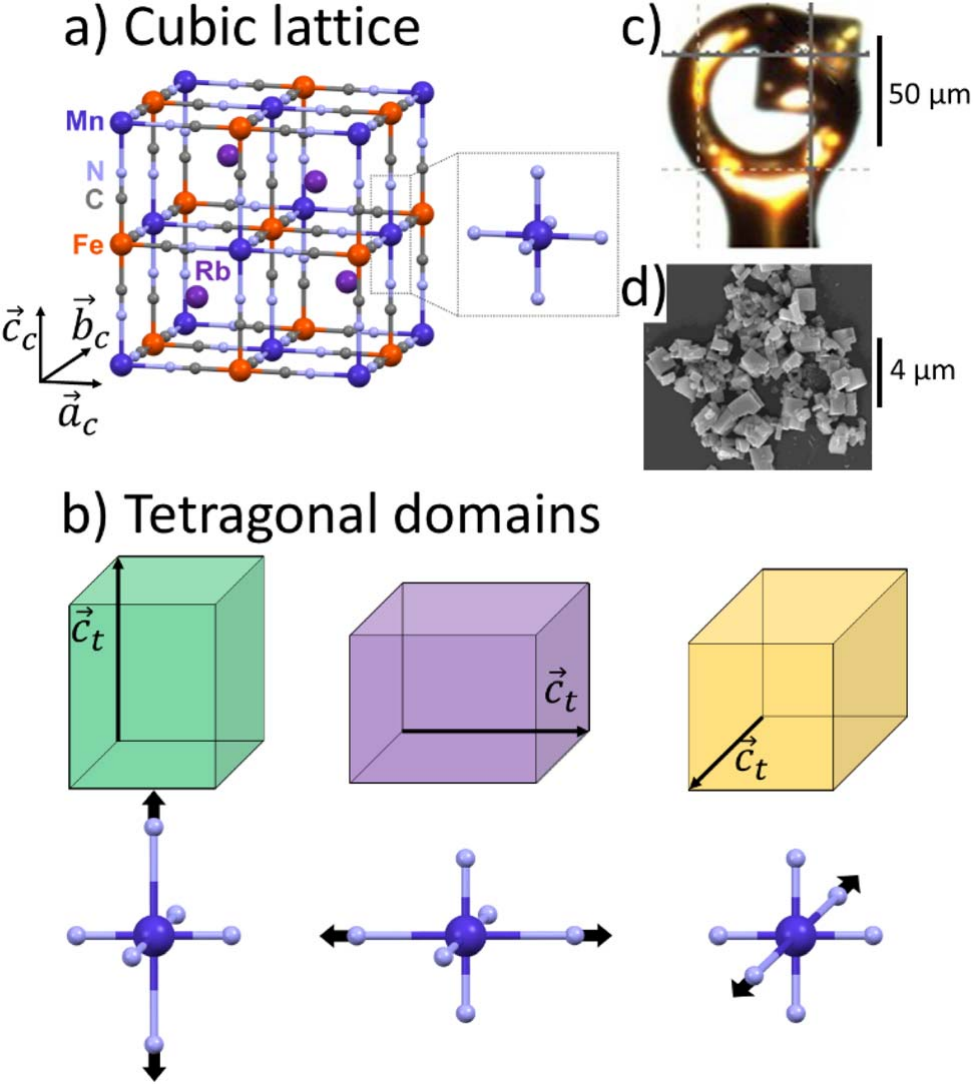
(a) Structure of RbMn[FeCN_6_] in HT Mn^II^Fe^III^ cubic lattice (*a*_c_ = *b*_c_ = *c*_c_), with MnN_6_ sites of *O*_h_ symmetry. (b) Tetragonal domains in Mn^III^Fe^II^ LT phase (*a*_t_ = *b*_t_ ≠ *c*_t_), shown in green, purple and orange, with 3 possible orientations of the tetragonal axis *c*_t_ along *a*_c_, *b*_c_ or *c*_c_ due to the Jahn–Teller distortion around the Mn^III^N_6_ sites with *D*_4h_ symmetry. (c) Image of the single crystal. (d) Scanning Electron Microscopy image of the powder.

Rubidium manganese hexacyanoferrate materials (Fig. 1a), with composition Rb_*x*_Mn[Fe(CN)_6_]_(*x*+2)/3_·*z*H_2_O, exhibit CT phase transitions, between Mn^II^(*S* = 5/2)Fe^III^(*S* = 1/2) high temperature (HT) cubic phase and Mn^III^(*S* = 0)Fe^II^(*S* = 0) low-temperature (LT) tetragonal phase, characterized by a wide thermal hysteresis.^19,20,41,42^ A Landau theory study revealed that this phase transition results from the coupling to the volume strain of the CT instability and the ferroelastic cubic–tetragonal phase transition associated with the collective Jahn–Teller distortion around the Mn^III^ sites.^43^ Crystallographic studies investigated the important lattice changes between the HT cubic phase (*F*4̄3*m*, *a*_c_ ≃ 10.5 Å) and the LT phase described in the non-conventional *F*4̄2*m* tetragonal space group, *a*_t_ ≃ 10.0 Å, *c*_t_ ≃ 10.5 Å).^43,44^ The cubic → tetragonal ferroelastic distortion is associated with the loss of the three-fold rotational symmetry of the cubic lattice. The structural instability occurs at the *Γ* point of the Brillouin zone and the Jahn–Teller symmetry-breaking order parameter belongs to the bidimensional *E* representation of the *F*4̄3*m* point group. Fig. 1b shows the cubic → tetragonal distortion, associated with the Jahn–Teller deformation of the Mn^III^N_6_ core along the *c*_t_ axis in the tetragonal phase. This collective distortion can equally occur along the principal directions *a*_c_, *b*_c_ or *c*_c_ of the initially cubic lattice.^6,9,10,45^ Therefore, 3 equivalent ferroelastic tetragonal domains can form in the LT phase.

Unexpected spectroscopic changes also occur in the LT phase. IR spectroscopy revealed a broad IR band (≃50 cm^−1^) around 2100 cm^−1^, attributed to the splitting of the C–N stretching mode, while a sharp (≃10 cm^−1^) band is observed in the HT phase.^46,47^ Optical spectroscopy revealed a broad and intense band around 500 nm,^48–50^ identified as a Mn-centred d–d transition, which is supposed to be weak in the LT *D*_4h_ symmetry. This absorption band plays a key role for driving photoinduced phase transition in these materials.^17,44,47,48^ The origin of these unusual spectroscopic features was however not clearly explained so far. Hereafter we use single crystal and powder X-ray diffraction (XRD) to study the ferroelastic domains in the LT phase and estimate the spatial extension of the lattice deformation around the domain walls.

## Experimental

### Sample preparation

Single crystals of RbMn[Fe(CN)_6_] (hereafter referred to as RbMnFe) were synthesized by gradually diffusing 2 mL of an aqueous solution of MnCl_2_·4H_2_O (1.0 mol dm^−3^) and 2 mL of an aqueous solution of K_3_[Fe(CN)_6_] (1.0 mol dm^−3^) into 100 mL of an aqueous solution of RbCl (0.5 mol dm^−3^) over one week at 40 °C. RbMnFe is known to exhibit a wide thermal hysteresis between ≃230 K and ≃300 K.^51,52^ Rb_0.94_Co_0.06_Mn_0.94_[Fe(CN)_6_]_0.98_·0.2H_2_O, referred to hereafter as RbMn_0.94_Co_0.06_Fe, was synthesized according to the procedure reported in previous works,^17^ where additional characterization data can be found. Magnetic measurements on the powder indicate a broad bistability regime around room temperature (*T*_↓_ = 253 K, *T*_↑_ = 328 K), between the LT and HT phases. The powder sample was either cooled below *T*_↓_ to generate the LT phase, or warmed above *T*_↑_ to generate the HT phase. It was then investigated at room temperature (293 K), where both phases are stable. Two identical batches of powder sample were prepared and analysed separately using two different powder X-ray diffraction techniques.

### Single crystal X-ray diffraction

We used single-crystal X-ray diffraction to study the RbMnFe sample. The data were collected in the HT phase at 300 K and in the LT phase at 100 K. Single-crystal X-ray diffraction was conducted using a Rigaku XtaLAB Synergy-R/DW with monochromatic Mo K_α_ radiation (*λ* = 0.70930 Å). We used paratone N oil to mount the single crystal (50 μm large) on a Micro MountsTM holder (Fig. 1c). The data analysis and the reciprocal space reconstruction was performed with CrisAlisPro software of Rigaku.

### Conventional powder X-ray diffraction of RbMn_0.94_Co_0.06_Fe

Conventional powder X-ray diffraction data of the RbMn_0.94_Co_0.06_Fe sample were collected using a Rigaku Ultima-IV diffractometer with Cu K_α_ radiation (*λ* = 1.5418 Å, scanning range: 10–70°, scanning speed: 1° min^−1^, sampling width: 0.02°). The powder sample consists of plate-shaped crystals, with an average size of 0.9 ± 0.3 μm (Fig. 1d). The LT phase was prepared by cooling the powder in HT phase with liquid nitrogen. After the cooling, the LT phase was sustained at room temperature due to the existence of the thermal hysteresis over room temperature. The attribution of the Bragg peaks was based on previous analysis using Rietveld refinement.^17^

### Streaming powder X-ray diffraction of RbMn_0.94_Co_0.06_Fe

In the second approach, XRD measurements were performed at the XPP beamline of the LCLS X-ray Free-Electron Laser (X-FEL), using monochromatic X-rays (*λ* = 1.890 Å, Δ*E*/*E* ∼ 10^−4^).^53,54^ We used the streaming powder technique, which makes it possible to study ultra-fast and persistent photoinduced phase transitions.^17,23^ The RbMn_0.94_Co_0.06_Fe crystals were dispersed in ethanol with a 1 : 90 crystal-solvent weight ratio, and the solution was streamed through a 75 μm free-flowing jet to interact with the X-ray beam. The diffracted intensity was collected shot-to-shot using an epix10k2M detector^55^ in the transmission geometry, at the repetition rate of the LCLS X-FEL (120 Hz). Each 2D image was azimuthally integrated using pyFAI^56^ and normalized on the X-ray intensity. A typical number of 10 000 shots are subsequently averaged, over an acquisition time of about 100 s. The resulting pattern extends in the [0.5 Å^−1^; 3.6 Å^−1^] *Q*-range. It contains a broad scattering contribution due to the solvent, which is removed by subtracting the scattering pattern of pure ethanol measured in the same conditions. The shape of the diffraction peak(s) is then analysed following the same procedure as for static powder XRD.

## Results and discussion

**Fig. 2:**
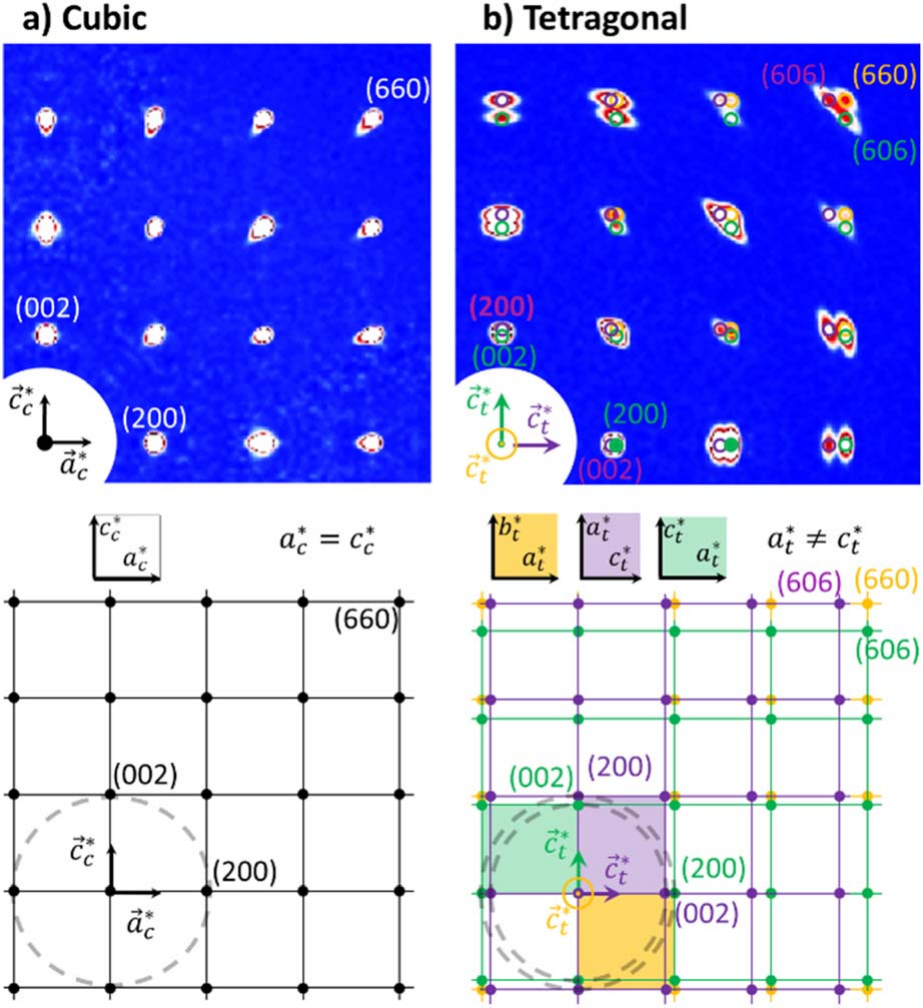
Single crystal X-ray diffraction data of RbMn[Fe(CN)_6_] (top) with a schematic drawing of the reciprocal lattice (bottom). (a) Diffracted intensity in the 
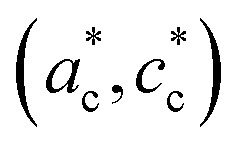
 plane with *k* = 0 for the cubic HT phase measured at 300 K, where the sharp Bragg peaks are located on the nodes of the cubic reciprocal lattice. (b) Diffracted intensity in the same reciprocal plane for the tetragonal LT phase (100 K), where the Bragg peaks are located on the nodes of the 3 superposing tetragonal reciprocal lattices of the 3 different domains (shown in orange, purple and green), characterized by different orientations of the tetragonal axis 
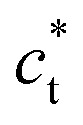
. Diffuse scattering appears around the broader Bragg peaks due to lattice distortions around the domain walls.

Fig. 2 shows single crystal XRD data, characteristic of the cubic → tetragonal phase transition of RbMn[Fe(CN)_6_]. The phase transition is associated with a symmetry breaking from HT cubic phase with *F*4̄3*m* space group (*a*_HT_ ≈ 10.5 Å) to LT non-conventional *F*4̄2*m* space group (*a*_LT_ ≈ 10.0 Å, *c*_LT_ ≈ 10.5 Å). In the cubic phase (Fig. 2a), the diffracted intensity in the (*h*0*l*) reciprocal plane, with cubic reciprocal lattice vectors 
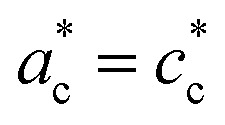
, corresponds to sharp Bragg peaks on the node where the *h*, *k* and *l* indices have same parity (F cell). The diffracted patterns are the same in the (0*kl*) and (*hk*0) planes due to cubic symmetry. In the LT phase (Fig. 2b), the lengths of the lattice vectors differ due to the tetragonal distortion 
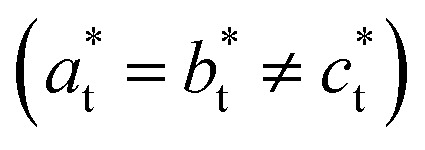
. We can see in Fig. 2b a splitting of the Bragg peaks due to the formation of the ferroelastic domains, for which the tetragonal distortion defining the 
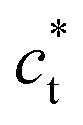
 axis can equally occur along the HT axes 
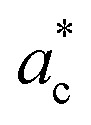
, 
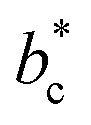
 or 
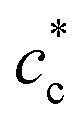
. The diffraction pattern in LT phase (Fig. 2b), corresponding to the (*h*0*l*) plane of HT phase (Fig. 2a), is then characteristic of the superposition of the diffracted signal from the 3 tetragonal domains with different orientations of the 
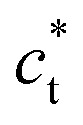
 axis in the LT phase: a square 
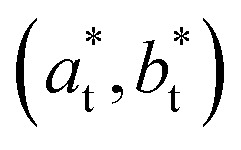
 lattice (yellow, with 
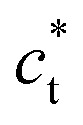
 perpendicular to the figure), and two rectangular 
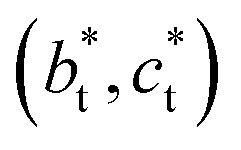
 and 
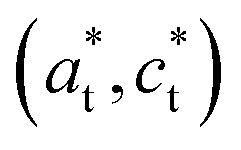
 lattices with 
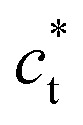
 vertical (green) or horizontal (purple). Consequently, the (*h*0*h*) Bragg peaks split in 3 peaks. We can also observe an anisotropic broadening of the Bragg peaks in the LT phase, with some diffuse scattering in-between the peaks of the different domains. This is particularly clear for the (606) Bragg peaks for example. This peak broadening is characteristic of non-uniform lattice strains.^57,58^

**Fig. 3:**
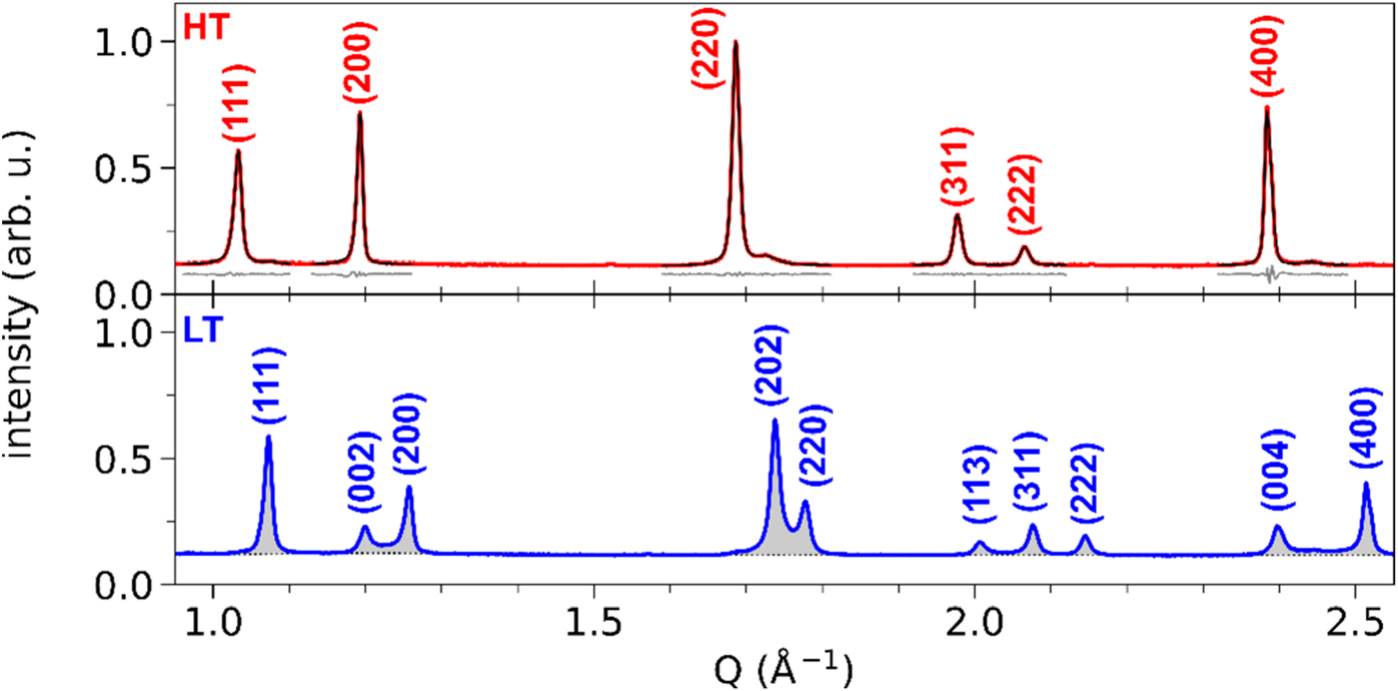
Conventional X-ray powder diffraction patterns of RbMn_0.94_Co_0.06_Fe in HT (red) and LT (blue) phases. (*hkl*) Miller indices correspond to the *F*4̄3*m* space group for the HT phase and to the *F*4̄2*m* space group for the LT phase. For the HT phase, the least-square fit of each peak is shown (black curve), together with the residuals (grey curve, shifted by +0.08). For the LT phase, only the fitted background is shown (black dotted line), highlighting the peak area (grey-shaded).

Fig. 3 shows the XRD powder diffraction patterns along the scattering vector *Q* of RbMn_0.94_Co_0.06_Fe, measured at room temperature in the HT and LT phases with conventional powder XRD. The most important changes in the diffraction pattern, due to the ferroelastic distortion in the LT phase, correspond to the splitting along scattering vector *Q* of the Bragg peaks (*h*00) and (00*h*), and of the Bragg peaks (*hh*0) and (*h*0*h*), which are initially equivalent in the HT phase. For instance, Fig. 2b shows that the (200) and (002) Bragg peaks are no longer located on the same diffraction ring defined by |*Q*| in the LT phase since |*Q*(200)| ≠ |*Q*(002)|. Compared to previous studies,^42,59^ the splitting of the (002) and (200) peaks, indexed the LT *F*4̄2*m* space group, corresponds to the splitting of the (200) and (110) peaks indexed the LT *I*4̄*m*2 space group. A similar splitting is observed for different Bragg peaks. In addition, due to lattice contraction, the peaks shift to higher *Q* in the LT phase. Fig. 3 shows asymmetric Bragg peaks in the LT phase, compared to HT, with diffuse scattering in between the peaks. As mentioned above, this is due to the lattice strains, also observed in single crystal XRD with the diffuse scattering around the Bragg peaks.

While conventional Rietveld refinement can be used to characterize uniform strain in powder patterns, it is usually inadequate to describe highly-anisotropic strains, that give asymmetric Bragg peaks.^60^ Instead, we used an approach similar to the one presented by Daniels *et al.* to characterize the domain structures from diffraction profiles in tetragonal ferroelastic ceramics.^61^ We analysed the shape of each peak or group of peaks separately by individual fitting, using a combination of split pseudo-Voigt peak profiles with a polynomial background:
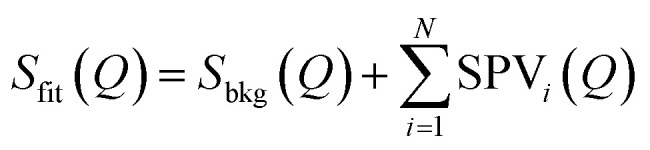
where *S*_bkg_(*Q*) is a second-order polynomial, and *N* is the number of fitted peaks (*N* ∈ [1; 3]). *S*_bkg_(*Q*) was fitted outside of the Bragg peak(s), and then fixed during the fit of the peak(s). The split pseudo-Voigt profile SPV(*Q*) is defined as an asymmetric peak shape, where the left (L) and right (R) sides around the peak centre *Q*_O_ are described as two distinct pseudo-Voigt functions:
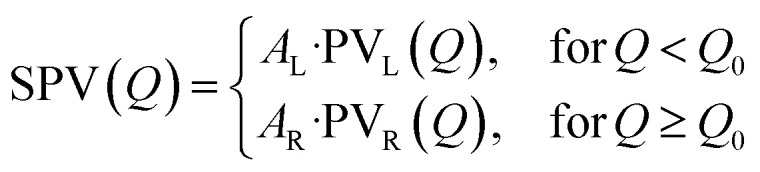
where PV_X_(*Q*) (X = L, R) is the usual combination of a Lorentzian and a Gaussian profile with a common half width at half maximum *σ*_X_ and a mixing fraction *α*_X_ ∈ [0; 1]:
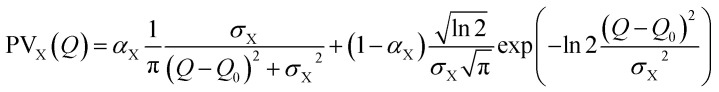
Finally, ensuring continuity at the peak centre *Q*_0_ imposes:
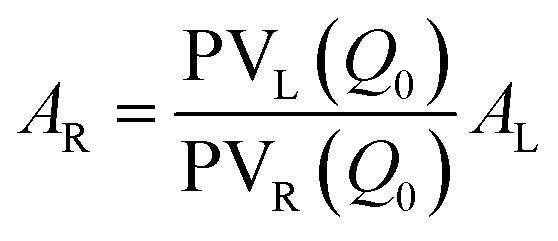


As such, SPV profiles enable the analysis of asymmetry in the peak shape profiles, by extracting separately the half widths and the peak shape on both sides of the peaks. In the following, the reported values of the parameters correspond to the best fit of the data, and the associated uncertainties are the standard deviations of the least-square fit. In the case of (111), (220) and (400) Bragg peaks, we had to consider a weak additional signal due to an impurity, the passive Rb_2_Mn^II^[Fe^II^(CN)_6_]·3.5H_2_O crystals (*Fm*3̄*m a* = 10.185(6) Å), known to form during the synthesis.^17^ This impurity does not exhibit CT nor ferroelastic phase transition and we used the same contribution from this impurity in the analysis of the signal of HT and LT states at 293 K.

**Fig. 4:**
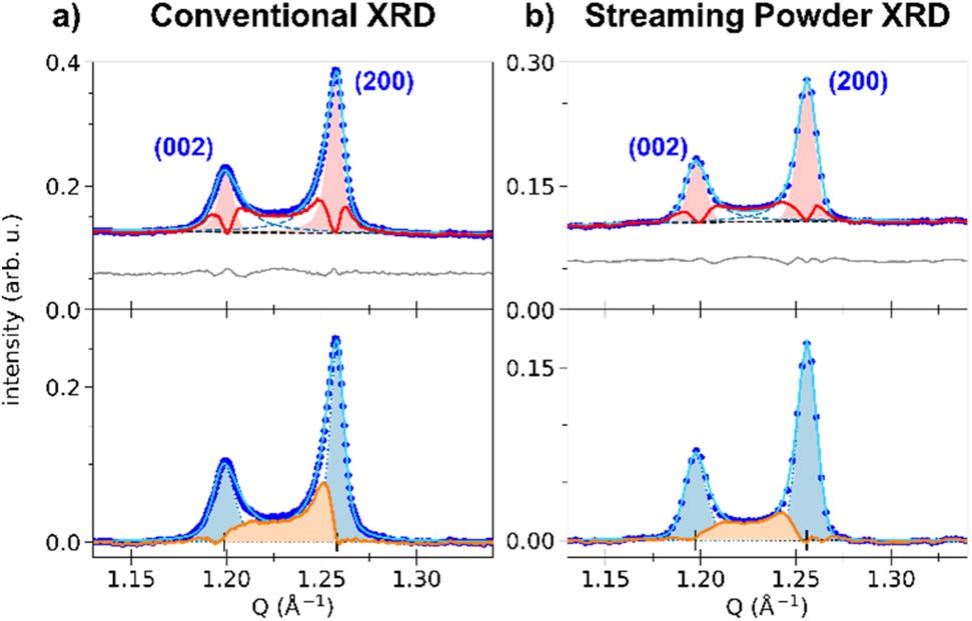
(a) Zoom of the LT conventional powder diffraction pattern on the (002) and (200) peaks (blue dots). (Top panel): the least-square fit is shown (light blue curve), together with the two SPV components describing each peak (blue dashed lines), the polynomial background (black dashed line) and the residuals (grey line, shifted by +0.06). For both peaks, the HT peak model is shown as the red-shaded area, and the associated differential between the LT pattern and the HT peak model is displayed as the red curve (see text). (Bottom panel): background-subtracted pattern, together with the fit (light blue curve), the symmetrized peak shapes (blue-shaded areas and blue dotted lines), and the strain-affected intensity (orange area). (b) Same analysis for monochromatic X-FEL diffraction, in the streaming powder configuration.

The top panel of Fig. 3 shows the least-square fit of all the Bragg peaks for the HT phase, where the SPV fit yields good results with quite symmetric profiles. For example, the fit of the (200) Bragg peak provides quite similar values for L/R sides: *σ*_L_ = 4.90(0.04) × 10^−3^ Å^−1^*vs. σ*_R_ = 3.88(0.03) × 10^−3^ Å^−1^ and *α*_L_ = 0.69(0.02) *vs. α*_R_ = 0.45(0.02). The same fitting procedure was applied to the LT phase. We focus first our attention on the (002) and (200) peaks. The top panel of Fig. 4a shows the fit with 2 SPVs, which is in good agreement with the XRD data. In this LT phase, the peak profiles are however strongly asymmetric, as characterized by the fitting parameters.

For the (002) Bragg peak, we obtained *σ*_L_ = 6.7(0.3) × 10^−3^ Å^−1^*vs. σ*_R_ = 10.3(0.3) × 10^−3^ Å^−1^ and *α*_L_ = 0.59(0.07) *vs. α*_R_ = 1 and for the (200) Bragg peak *σ*_L_ = 7.51(0.08) × 10^−3^ Å^−1^*vs. σ*_R_ = 4.39(0.08) × 10^−3^ Å^−1^ and *α*_L_ = 1 *vs. α*_R_ = 0.68(0.03). Compared to the HT phase, both LT peaks are broader (*σ*^LT^_L,R_ > *σ*^HT^_L,R_), and display a significant L/R asymmetry: the width of the peaks is larger in between the two Bragg peaks compared to their outer part (*σ*_R_^(002)^ > *σ*_L_^(002)^ and *σ*_L_^(200)^ > *σ*_R_^(200)^). The inner part of the two peaks also have a more pronounced Lorentzian character, as *α*_X_ reaches its maximum value (1). This fitting procedure thus maps the non-negligible intensity observed between the two peaks in the LT phase, in agreement with Fig. 2b. This asymmetry of the powder diffraction peaks is well understood from the single crystal data in Fig. 2b, as diffuse scattering is observed at |*Q*| in between |*Q*(200)| and |*Q*(002)|. The present fit shows that this intensity cannot be explained by a simple isotropic broadening of the peaks as observed in other ferroelastic materials.

We can highlight the asymmetry of the LT (002) and (200) peaks by comparing their profiles with the more symmetric one of the HT (200) peak. The LT peaks were fitted with the SPV profile of the HT phase, with widths *σ*_L,R_ and fractions *α*_L,R_ fixed to the HT values and refined amplitudes and centres. The result is shown in Fig. 4a, where the red-shaded areas corresponds to fit of the peaks with the HT profiles. The differential between the experimental XRD pattern and the result from this fit is shown by the red curve. It is clear that the parameters of the sharp HT Bragg peaks poorly reproduce the LT data, as only the maximum of each peak is well described. The differential curve between the fit and the experimental data highlights the strong XRD signal between the (002) and (200) Bragg peaks, their asymmetry and broadening. Interestingly, a differential signal is also observed on the outer part of the two Braggs (*i.e.*, below 1.20 Å^−1^ and above 1.26 Å^−1^).

Thus, the LT Bragg peaks are globally broadened compared to the HT phase and the signal observed in between (002) and (200) is characteristic of lattice strains in the LT phase, due to the formation of ferroelastic domains walls in the crystal.

**Fig. 5:**
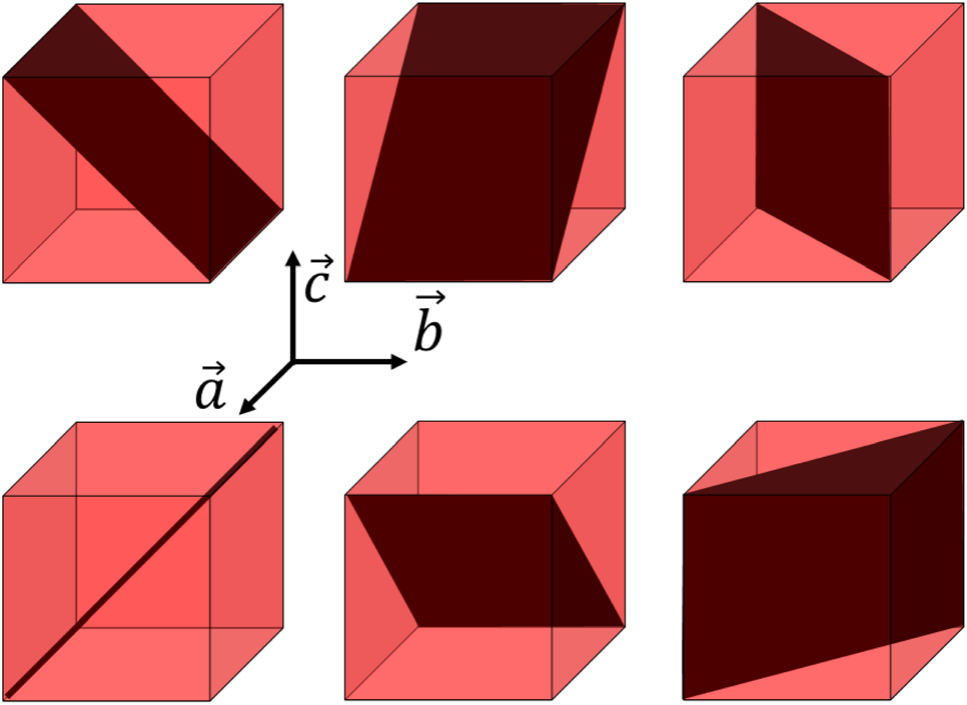
Domain walls in a cubic lattice with tetragonal distortion, corresponding to {101} planes or symmetry-equivalent.

Indeed, the strong asymmetry of the (200) and (002) Bragg peaks is due to the fact that tetragonal crystals have 90° {101}-type ferroelastic domain walls.^62–65^ There is an important strain due to the difference between the LT lattice parameters, with *a*_t_ ≃ 10.0 Å and *c*_t_ ≃ 10.5 Å. The different possible orientations of the {101}-type domain walls are shown in Fig. 5.

**Fig. 6:**
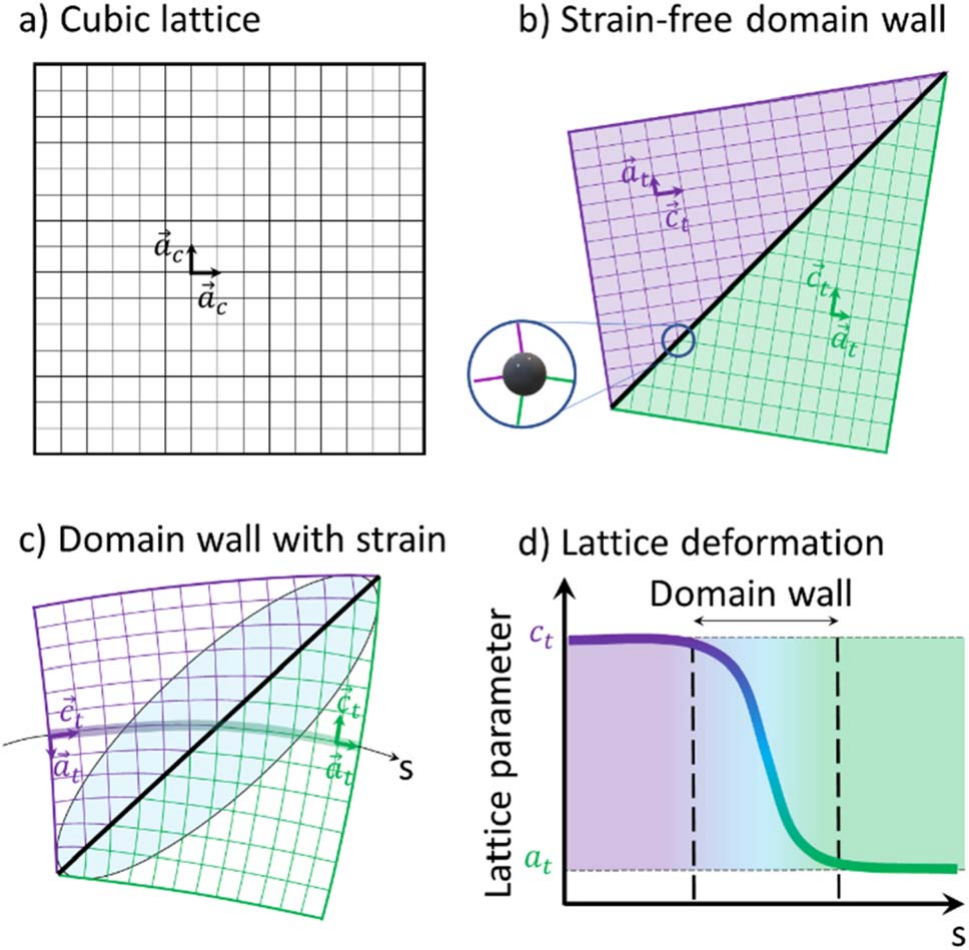
Schematic representations of the cubic lattice (a) in the HT phase, and two tetragonal domains in the LT phase (b) with the tetragonal axis *c*_t_ horizontal (purple) or vertical (green) for a strain-free domain wall. The inset shows the distortion of the metallic sites on the wall. (c) Domain wall with strained region represented by the blue shaded elliptical area. (d) Evolution of the lattice parameter through the domain wall.

Fig. 6 shows a 2D representation of the formation of two ferroelastic domains. Compared to the regular HT cubic lattice (Fig. 6a), tetragonal domains can form in the LT phase, separated by a wall. The geometrically strain-free twinning angle only depends on the *c*_t_/*a*_t_ ratio (Fig. 6b). However, due to constraints during the cubic-to-tetragonal phase transition and the development of the domains, this ideal twinning angle is not achieved. This is especially true in this polymeric crystal as the metallic sites, including the ones on the wall, are stabilized in *D*_4h_ symmetry (Fig. 1b). The sites on the wall for the strain-free twinning strongly deviates from *D*_4h_ symmetry as shown by the inset in Fig. 6b, which costs too much energy. Therefore, the lattice has to deform as shown in Fig. 6c, which results in strain fields in the vicinity of domain walls. The lattice constant changes gradually from *c*_t_ to *a*_t_ as we cross the domain wall along the path *s* shown in Fig. 6c, which represents the Mn–N–C–Fe bridges of the lattice. This change of lattice parameter is schematically shown in Fig. 6d. These strain fields around the wall, corresponding to a gradient of lattice parameter, result in the broadening of the Bragg peaks and diffuse scattering around some of them. The volume of material close to the domain wall that is affected by this strain field depends on the elastic modulus of the material. In the powder pattern, the signal between (002) and (200) is a direct signature of this strain field and the shape and amplitude of this inner signal reflects the strains extending around the domain walls.

To further characterize the gradient of lattice parameters around the domain walls, we performed a strain analysis, with an approach similar to the one proposed by Daniels *et al.*^61^ In the hypothetical absence of gradient, both (002) and (200) peaks would display symmetric profiles. Thus, we artificially constructed symmetrized peak profiles *S*_symm_(*Q*), using the fit parameters of the outer parts of the two peaks, which is not affected by the gradient:*S*_symm_^(002)^(*Q*) = *A*_L_^(002)^·PV_L_^(002)^(*Q*)*S*_symm_^(200)^(*Q*) = *A*_R_^(200)^·PV_R_^(200)^(*Q*)

The X-ray diffraction signal affected by the strain of the gradient can then be calculated by subtracting the symmetrized peak profiles:*S*_strain_(*Q*) = *S*(*Q*) − [*S*_symm_^(002)^(*Q*) + *S*_symm_^(200)^(*Q*)]

The resulting signal is shown as the orange curve in Fig. 4a. As can be seen, the signal spreads over the whole *Q* range between the two peaks. Its shape is similar to the expected strain distribution around domain walls, as discussed in the literature for other ferroelastic phase transitions.^61^ A maximum is observed on the upper *Q* limit, that can be attributed to difference in the peak multiplicity between (*h*00) and (00*h*) Bragg peaks in the tetragonal lattice. Furthermore, this analysis enables to estimate the total volume of the crystals that is affected by the strain, by integrating *S*_strain_(*Q*) and comparing it to the total integrated signal. In the present case, our fit indicates that the strain-affected volume represents ∼30% of the crystalline volume. This means that the formation of the ferroelastic domain walls induces important strains, which extend over about one third of each crystal.

Additionally, the width of the outer part of (002) and (200) peaks brings additional signature of the presence of ferroelastic domains. The outer parts of these peaks are indeed not affected by the gradient of the domain walls, and their broadening compared to the HT model cannot be related to lattice strain from *c*_t_ to *a*_t_. Instead, the larger width is due to the small size of the ferroelastic domains forming during the HT-to-LT phase transition. Indeed, as domains are created in the LT phase, the coherence length of the diffraction signal decreases from the crystal size in the HT phase to the domain size in the LT phase. The formation of small domains increases the width of the peaks, according to the Scherrer equation:
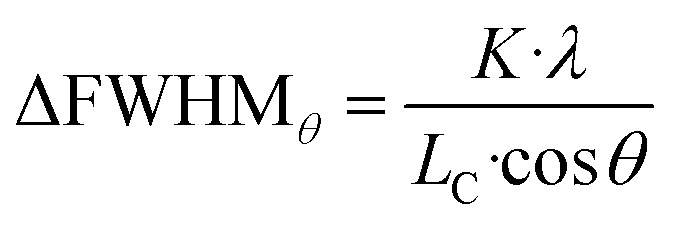
where ΔFWHM_*θ*_ is the full width at half maximum (FWHM) of the peak, corrected from the instrument response function (IRF), *K* is the shape factor of the order of unity, *λ* is the X-ray wavelength, *L*_C_ is the coherence length, and *θ* the diffraction angle of the Bragg peak.

We can therefore estimate the domain size from the broadening of (002) and (200) Bragg peaks relative to the HT phase, by considering that the width of HT (200) peak is mainly due to the IRF and that only the small size of the domains is responsible for the peak broadening in the LT phase. In this way, the increase of width of the LT (002) and (200) peaks, compared to HT (200) peak, gives ΔFWHM_*θ*_^(002)^ and ΔFWHM_*θ*_^(200)^. Given that we considered pseudo-Voigt peak profiles, ΔFWHM_*θ*_ was estimated as a mixture of Gaussian- and Lorentzian-corrected FWHMs:
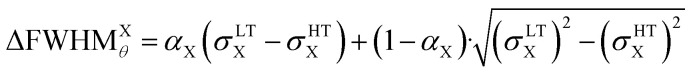
In this way, we find that *L*_c_ is within the range of 200–600 nm. This means that the size of the ferroelastic domains is of the order of several hundreds of nanometers. Since the average size of the crystals in the powder is typically 900 nm,^17^ crystals are composed of a few to tens of domains with few 100 s nm size.

These signatures of ferroelastic domains were further confirmed by streaming powder X-ray diffraction measurements on RbMn_0.94_Co_0.06_Fe at the XPP beamline at LCLS (Fig. 4b). Similar trends are measured, with asymmetric peak shapes and peak broadening. Slight differences are visible in the shape of the strain-affected signal, whose maximum is less pronounced, and with a strain-affected volume of ∼20%. This difference, compared to Fig. 4a, may be due to the batch used or to the different thermal cycling of the samples during the experiments. Nonetheless, the overall signatures of the strain around the ferroelastic domain walls are clearly observed.

**Fig. 7:**
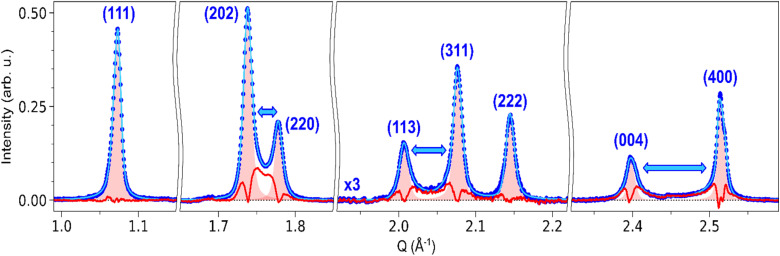
Fit of (111), (202)/(220), (113)/(311)/(222) and (004)/(400) Bragg peaks in the LT phase: background-subtracted pattern (blue dots), least-square fit with SPV functions (light blue curve), HT peak models corresponding to each peak (red-shaded area), and the associated differential between the LT pattern and the HT peak model (red curve). For the (113)/(311)/(222), the signal is magnified by a factor of 3 for visibility. Blue arrows indicate the peaks that are split in the LT phase due to the phase transition. In the specific case of the fit of (004)/(400), SPV functions are replaced with split Pearson VII distributions, to account for the large splitting between the two peaks.

A similar influence of the strain around the domain walls is also expected for other Bragg peaks. Fig. 7 shows the fit of (111), (202)/(220), (113)/(311)/(222) and (004)/(400) peaks, and their comparison with the corresponding HT model. The profile of the (111) peak matches the HT model, as indicated by a negligible differential. This is also true for the (222) peak. On the other hand, (202)/(220), (113)/(311) and (004)/(400) all display a behaviour similar to (002)/(200): strong differential scattering intensity is observed between the peaks, as well as broadening on the outer part of the peaks. These three groups of peaks indeed correspond to peak splitting due to the ferroelastic phase transition, as (002)/(200). The gradient imposed by the domain walls during the transition thus affects the corresponding lattice constant. However, compared to the seminal case of (002)/(200) splitting, further strain analysis is difficult for these peaks, since their broadening can be affected on both left and right sides by the strain around the domain walls. For instance, (202) is affected by the *a*_LT_ → *c*_LT_ gradient on the left side, and by the *c*_LT_ → *a*_LT_ gradient on the right side. In the case of (111) and (222), the good fit with the HT model shows that the strain field is weaker along these crystalline directions, as expected for {101}-type ferroelastic domain walls.

## Conclusions

This X-ray diffraction study of the ferroelastic phase transition from the cubic Mn^II^Fe^III^ HT to the tetragonal Mn^III^Fe^II^ LT phase of RbMnFe materials reveals the formation of ferroelastic domains walls during the cooperative Jahn–Teller distortion around the Mn sites. Three types of ferroelastic tetragonal domains, with *c*_t_ axis pointing in all three possible directions, coexist in the LT phase, as revealed by single-crystal X-ray diffraction. Our analysis shows that the spatial extension of the strain around the domain walls represents ≃30% of the volume. Because of these distortions of the polymeric Mn–N–C–Fe network, the local symmetry of the ligand field of the metal ions around the wall deviates from *D*_4h_, as shown in Fig. 6c. In this figure, a single domain wall is represented. However, as explained in Fig. 5, different domain walls with different orientations can form. Along the path *s* in Fig. 6c, the *c*_t_ axis points up, while the *a*_t_ axis points down. However, around a symmetry-equivalent wall the *c*_t_ axis will point down, while the *a*_t_ axis will points up. In average, *c*_t_ and *a*_t_ axes are horizontal. The angular distribution of these axes broadens transversally the Bragg peaks (Fig. 2b). Our analysis, evidences that a large fraction of the sample (≃30% of the volume) is affected by the lattice strain around the domain wall. This translates through the appearance of scattering signal in-between Bragg peaks, such as (002)/(200) for example. Such a diffuse signal was observed in previous experiments, but strain analysis was never performed so far. Our strain analysis makes it possible to understand the unexpected spectroscopic changes occurring in the LT phase. On the one hand, a broad IR band (≃50 cm^−1^) of the C–N stretching mode is observed in the LT phase, compared to the sharp IR band (≃10 cm^−1^) in the HT phase.^47^ This broadening results from both a splitting of this IR band due to the symmetry-breaking in the tetragonal phase and a broadening of these bands due to the strain field around the wall. On the other hand, a broad and intense optical band, which has a strong Mn-centred d–d character, appears around 500 nm in the LT phase,^48^ which is not expected for Mn sites in *D*_4h_ symmetry. Our results show that about 30% of the Mn sites are in a strong strain field. Consequently, the associated lattice strain, bending the Mn–N–C–Fe bridges, activates this optical transition. The strain field around domain walls plays therefore an important role for the optical control of the magnetic properties of RbMnFe materials, as the Mn-centred d–d transition is also used to drive photomagnetic properties.^17,44,47,48^ This highlights the important consequences of the formation of a domain wall. The present strain analysis is thus complementary to the structural information obtained though, *e.g.*, Rietveld refinements. Our next goal is to extend this study to the time domain, to understand the evolution of lattice strains during ultrafast and persistent photoinduced phase transition.^17^ This family of multifunctional materials^19^ has good chemical stability and durability, and does not contain any banned elements, making it a promising candidate for practical applications, such as barocaloric effects,^66^ photomagnetism,^20,41,67^ or Ferroelectricity.^68^ It was also shown that the application of an electric field above a threshold value leads to a transition from the high- to the low-temperature phase^69^ and consequently changes in their magnetic, optical, and electronic properties. This can open the way for novel electro-optical devices, and here again the domain walls may also play an important role in the process.

## Data availability

The powder X-ray diffraction data and their fit are available at Zenodo https://doi.org/10.5281/zenodo.13366596.

## Author contributions

Marius Hervé: methodology, data curation, formal analysis, writing – original draft; Shintaro Akagi: resources, data curation, investigation, formal analysis; Laurent Guérin, formal analysis; Leland Gee: data curation; Ryan Ribson: data curation; Matthieu Chollet: data curation; Marco Cammarata: data curation, funding acquisition; Shuntaro Nagashima: data curation, investigation, formal analysis; Shin-ichi Ohkoshi: funding acquisition, supervision; Hiroko Tokoro: resource, data curation, funding acquisition, supervision; Eric Collet: funding acquisition, data curation, investigation, conceptualization, supervision, writing – original draft, review & editing. All authors critically reviewed and revised the manuscript draft and approved the final version for submission.

## Conflicts of interest

There are no conflicts to declare.
